# Ciprofol versus propofol for painless gastrointestinal endoscopy: a dose-stratified systematic review and meta-analysis

**DOI:** 10.3389/fphar.2026.1977897

**Published:** 2026-09-25

**Authors:** Jing Liu, Jun Yu, Zhiyong Chen, Yuchao Bai, Ximeng Zhang, Jian Du

**Affiliations:** 1 Department of Pharmacy, Tianjin Beichen District Traditional Chinese Medicine Hospital, Tianjin, China; 2 Department of Physical Examination, Tianjin Beichen District Traditional Chinese Medicine Hospital, Tianjin, China; 3 Department of Anaesthesiology, Tianjin Beichen District Traditional Chinese Medicine Hospital, Tianjin, China

**Keywords:** ciprofol, dose, meta-analysis, painless gastrointestinal endoscopy, propofol

## Abstract

**Background:**

We compared ciprofol with propofol for painless gastrointestinal endoscopy, focusing on dose, recovery, induction, satisfaction, and adverse events.

**Methods:**

PubMed and Embase were searched through May 2026 for randomized controlled trials in adults undergoing painless gastrointestinal endoscopy. We pooled weighted mean differences (WMDs) for continuous outcomes and risk ratios (RRs) for dichotomous outcomes using random-effects models. Risk of bias was assessed with the Cochrane tool. The protocol was prospectively registered in PROSPERO (CRD420251088249).

**Results:**

Eleven trials (2,084 participants) met the criteria. Recovery was statistically longer with ciprofol (WMD 0.83 min, 95% CI 0.16 to 1.49; P = 0.01), but the absolute difference was under 1 min; the difference was observed at 0.4 and 0.5 mg/kg. Induction time and physician and patient satisfaction did not differ significantly. Ciprofol was associated with fewer reported episodes of hypotension (RR 0.81, 95% CI 0.72–0.90), injection pain (RR 0.13, 95% CI 0.08–0.22), respiratory depression (RR 0.47, 95% CI 0.27–0.83), and hypoxemia (RR 0.64, 95% CI 0.42–0.96).

**Conclusion:**

Ciprofol and propofol produced broadly comparable procedural outcomes. The apparent advantages for selected adverse events and the dose-related recovery pattern are uncertain because all trials were conducted in China, several pooled outcomes were highly heterogeneous, outcome definitions varied, and publication bias was possible. The dose-stratified findings are exploratory, and the results are hypothesis-generating and require confirmation in larger international trials.

**Systematic Review Registration:**

https://www.crd.york.ac.uk/PROSPERO/view/CRD420251088249, identifier PROSPERO CRD420251088249.

## Introduction

Endoscopic examination is central to the diagnosis and management of gastrointestinal disease (Xie et al., 2025; Liao et al., 2023), and the continuing global burden of gastrointestinal malignancies makes timely detection especially important (Bray et al., 2024). It can also support symptom control and treatment planning in advanced disease (Arnold et al., 2020; El Hajjar and Rey, 2020). However, an unsedated procedure may cause gagging, pain, anxiety, and other distress that interfere with cooperation and may increase physiologic stress (Zheng et al., 2022; Zhang et al., 2016). Current professional guidance supports appropriate sedation to improve patient tolerance and procedural conditions (Early et al., 2018). Selecting a sedative therefore requires a balance between reliable procedural conditions and rapid recovery, while minimizing respiratory, hemodynamic, and injection-related complications (Cheng et al., 2025; Mi et al., 2023).

Propofol is widely used for endoscopic sedation because it acts quickly, has a short half-life, and is cleared efficiently (Zhou S. et al., 2025). Its principal pharmacologic effect is enhancement of inhibitory signaling through the γ-aminobutyric acid (GABAA) receptor (Shin et al., 2022) (Sahinovic et al., 2018). These properties make it effective, but dose-related hypotension, bradycardia, and respiratory depression remain clinically relevant concerns (Zhou J. et al., 2025; Li et al., 2022; Yang et al., 2025) (Liu X. et al., 2023). Injection pain is also common and may worsen the patient’s experience of induction (Teng et al., 2021). Although uncommon, propofol infusion syndrome can cause metabolic derangement and multiorgan failure and can be fatal (Wang et al., 2025).

Ciprofol is a newer intravenous anesthetic in the 2,6-disubstituted phenol class. It acts at the GABAA receptor, like propofol, but its pharmacologic profile differs from that of propofol (Lu et al., 2023). Available evidence suggests more favorable pharmacokinetic and pharmacodynamic characteristics (Bajwa et al., 2023). The cyclopropyl substitution increases lipophilicity and supports penetration into the central nervous system (Zhou J. et al., 2025; Hung et al., 2023). Ciprofol also shows greater receptor selectivity and affinity than propofol (Lu et al., 2023), and its reported potency is approximately four-to fivefold higher, allowing comparable sedation at a lower dose (Liu SB. et al., 2023). Clinical studies have further associated ciprofol with less hemodynamic and respiratory disturbance and less injection pain, although these advantages require confirmation across settings (Petkar et al., 2024).

Clinical trials and earlier evidence syntheses have compared these drugs (Liao et al., 2023; Li et al., 2022; Teng et al., 2021), but the relationship between the initial ciprofol dose and recovery or induction outcomes has not been consistently examined. We therefore designed this systematic review and meta-analysis to compare ciprofol with propofol during painless gastrointestinal endoscopy, focusing on dose-stratified recovery and induction outcomes, satisfaction, and adverse events.

## Methods

This systematic review and meta-analysis was reported in accordance with PRISMA 2020 (Page et al., 2021). The protocol was prospectively registered in PROSPERO (CRD420251088249). No separate public protocol was available; the PROSPERO record provides the registration information. The completed PRISMA 2020 checklist is provided as Supplementary File 1.

### Search strategy

We searched PubMed and Embase from database inception to May 2026. The electronic strategy combined ciprofol-related terms (“Ciprofol,” “HSK3486,” and “Cipepofol”) with propofol-related terms (“Propofol,” “Diprivan,” “Disoprofol,” “Disoprivan,” “Aquafol,” and “Fresofol”). The complete database-specific strategies are reproduced in Supplementary Table 1. Reference lists of eligible reports were also screened. We did not conduct a separate grey-literature search.

### Eligibility criteria

Eligibility was defined in advance using PICOS. The population was adults undergoing painless gastrointestinal endoscopy, including gastroscopy, colonoscopy, or combined gastrointestinal endoscopy. The intervention was ciprofol, used alone or with an opioid agonist; the comparator was propofol, used alone or with an opioid agonist. Eligible studies reported extractable data for at least one prespecified outcome: recovery time, induction time, physician satisfaction, patient satisfaction, hypotension, injection pain, bradycardia, respiratory depression, hypoxemia, nausea, or vomiting. Only randomized controlled trials were eligible.

We excluded records that were clearly outside the topic at title or abstract screening, as well as reviews, case reports, conference abstracts, meta-analyses, and letters. We also excluded non-English reports, non-randomized studies, studies of children or other non-adult populations, and studies that did not evaluate gastroscopy, colonoscopy, or gastrointestinal endoscopy. Two reviewers (J.L. and J.Y.) independently screened titles/abstracts and then full texts. EndNote’s duplicate-detection function was followed by manual checking. Disagreements were settled by discussion, with Z.C. acting as the third reviewer when needed.

### Risk-of-bias assessment

Two reviewers (Y.B. and X.Z.) independently judged study quality with the Cochrane Risk of Bias Tool (Higgins et al., 2019). The assessment covered random-sequence generation, allocation concealment, blinding of participants and personnel, blinding of outcome assessors, incomplete outcome data, selective reporting, and other potential sources of bias. Each domain was rated as low, high, or unclear risk. Differences were discussed and referred to Z.C. when consensus was not reached.

### Data extraction

J.L. and J.Y. independently extracted information from each eligible full text using a predefined form. Extracted study-level variables included author, publication year, country, design, outcomes, comparator, and ciprofol dosing; initial doses were recorded, whereas supplemental boluses or maintenance regimens were recorded when available. Patient-level variables included body mass index, ASA class, histories of hypotension, diabetes, and cardiovascular or cerebrovascular disease, mean age and standard deviation, sex distribution, sample size, and endoscopy type. Because supplemental dosing was inconsistently reported, it was neither pooled nor used as a dose-stratification factor.

Any discrepancy in extraction was discussed until the reviewers reached agreement, with Z.C. arbitrating unresolved issues. When a report did not provide necessary information, we attempted to contact its corresponding author by email.

### Outcome measures

We defined recovery time as the interval from eye opening to an Aldrete score of at least 9 (Li et al., 2024), and induction time as the interval from administration of the study drug to a MOAA/S score of one or less (Li et al., 2024). These were the primary outcomes; the definitions represent recovery sufficient for discharge and sedation adequate to begin a painless endoscopic procedure, respectively. Recovery time and induction time were defined as described above and these were treated as reference definitions. The definitions were not identical across trials: sedation depth was most often assessed with the MOAA/S (target ≤1 in most trials, but three in one trial and the Narcotrend index in another), and recovery was variously reported as time to an Aldrete score ≥9, time to full alertness (MOAA/S 5), awakening time, or time to discharge. Where a trial used a different metric, the metric reported by that trial was analysed, and this variation is considered when interpreting the pooled estimates.

Secondary outcomes were physician and patient satisfaction, measured with the scales or questionnaires used in each trial. We also extracted hypotension, injection pain, bradycardia, respiratory depression, hypoxemia or low oxygen saturation, nausea, and vomiting. For adverse events, we retained each study’s operational definition. Because the thresholds for hypotension, respiratory depression, and hypoxemia were not uniform across trials, the pooled safety estimates are reported as differences in the rates of reported events rather than as established safety advantages, and this is stated explicitly in the Abstract, Results, Discussion, and Conclusions.

### Statistical analysis

For continuous outcomes, we calculated weighted mean differences (WMDs) with 95% confidence intervals (CIs); for dichotomous outcomes, we calculated risk ratios (RRs) with 95% CIs. Statistical heterogeneity was evaluated with Cochran’s Q and quantified with the I^2^ statistic (I^2^ = 100% × (Q − df)/Q) and H^2^ (H^2^ = Q/df) (Huedo-Medina et al., 2006); between-study variance was estimated with restricted maximum likelihood (τ^2^). We used random-effects models because differences in drug dosing and procedure type were expected. Leave-one-out analyses assessed the influence of individual studies. Prespecified subgroup analyses and meta-regression examined ciprofol dose and endoscopy type; age, ASA class, co-administered opioids, and supplemental dosing were not modeled because reporting was incomplete or inconsistent. Funnel plots and Egger’s test were used to explore small-study or publication bias, and trim-and-fill was applied as a sensitivity analysis when indicated. P < 0.05 was considered statistically significant. Analyses were conducted in Stata 17.0; forest plots were generated and the shared-comparator split analysis described below and the GRADE assessment were performed in Python 3.12. Two trials compared more than one ciprofol dose with a single propofol group. To avoid counting participants in the shared comparator group more than once, the shared propofol group was divided evenly across the corresponding dose comparisons, and this split analysis was used as the primary analysis for every outcome affected by a shared comparator. For continuous outcomes, the sample size of the shared comparator was divided by the number of comparisons, with the reported mean and standard deviation left unchanged; for dichotomous outcomes, both the number of events and the number of participants in the shared comparator were divided by the number of comparisons. Outcomes to which no multi-arm trial contributed were unaffected. In the primary (split) analysis, recovery time remained 0.83 min longer with ciprofol (95% CI 0.16–1.49).

### Ethics statement

This is a systematic review and meta-analysis, ethics approval and consent to participate are not applicable.

## Results

### Literature search and study selection

The database searches and reference-list screening yielded reports comparing ciprofol and propofol at different initial dose levels for painless gastrointestinal endoscopy.

Eleven randomized controlled trials met all eligibility criteria (Liao et al., 2023; Li et al., 2022; Teng et al., 2021; Li et al., 2024; Chen et al., 2023; Zhang et al., 2024; Zhou et al., 2024; Chen et al., 2022; Gao et al., 2024; He et al., 2024; Liu Y. et al., 2024). The study-selection pathway is shown in the PRISMA flow diagram (Figure 1).

**FIGURE 1:**
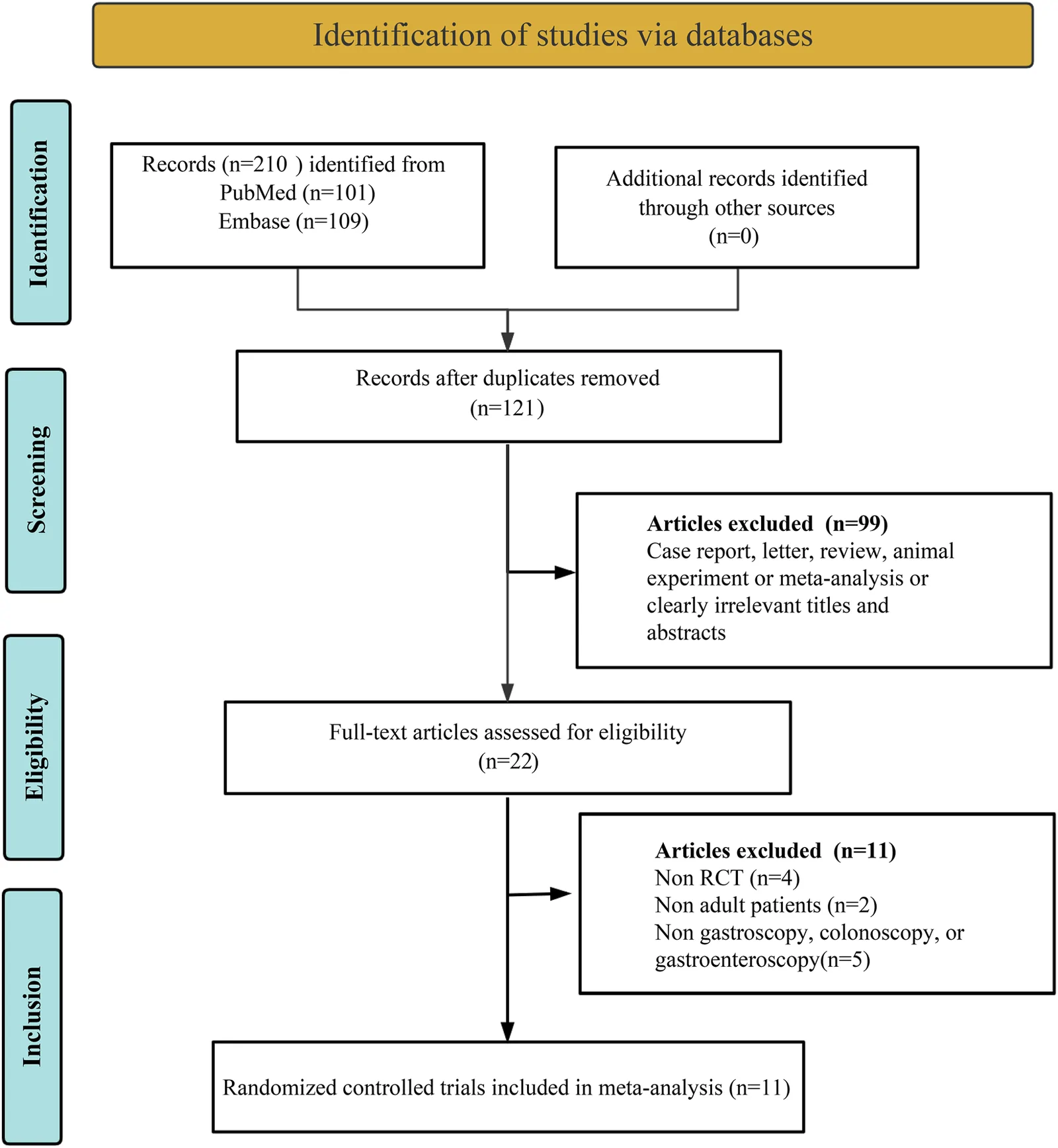
PRISMA flow diagram showing identification, screening, eligibility assessment, and inclusion of randomized trials comparing ciprofol with propofol in painless gastrointestinal endoscopy.

### Study characteristics and risk of bias

Across the 11 trials, 2,084 participants were included; individual sample sizes ranged from 60 to 368. All studies were conducted in China and were published from 2021 to 2024. Initial ciprofol doses ranged from 0.2 to 0.5 mg/kg: 0.2 mg/kg was evaluated in one study (Chen et al., 2023), 0.3 mg/kg in three (Chen et al., 2023; Zhang et al., 2024; Zhou et al., 2024), 0.4 mg/kg in eight (Liao et al., 2023; Li et al., 2022; Teng et al., 2021; Chen et al., 2023; Zhou et al., 2024; Chen et al., 2022; Gao et al., 2024; He et al., 2024), and 0.5 mg/kg in four (Teng et al., 2021; Li et al., 2024; Zhou et al., 2024; Liu Y. et al., 2024). Two studies enrolled only gastroscopy patients (Li et al., 2024; Liu Y. et al., 2024), three enrolled only colonoscopy patients (Teng et al., 2021; Gao et al., 2024; He et al., 2024), and three included broader gastrointestinal endoscopy populations (Zhang et al., 2024; Zhou et al., 2024; Chen et al., 2022). Most participants were classified as ASA I or II, but age and ASA reporting was incomplete and did not support further subgroup analyses. Study and participant characteristics are summarized in Tables 1 and 2.

**TABLE 1:** Characteristics of the included trials.

Author	Year	Country	Study design	Outcome	Comparison	Initial dose (mg/kg)
Chen et al.	2023	China	RCT	(2) (4) (6) (10)	Ciprofol group	0.2 mg/kg
					Ciprofol group	0.3 mg/kg
					Ciprofol group	0.4 mg/kg
					Propofol group	1.5 mg/kg
Chen et al.	2022	China	RCT	(2) (5) (6) (7) (8)	Ciprofol group	0.4 mg/kg
					Propofol group	1.5–2.0 mg/kg
Gao et al.	2024	China	RCT	(1) (2) (3) (4) (5) (6) (7) (8) (10)	Ciprofol group	0.4 mg/kg
					Propofol group	2.0 mg/kg
He et al.	2024	China	RCT	(1) (2) (3) (5) (6) (7) (8) (9) (10)	Ciprofol group	0.4 mg/kg
					Propofol group	2.0 mg/kg
Li et al.	2022	China	RCT	(1) (2) (3) (4) (5) (6) (8) (9)	Ciprofol group	0.4 mg/kg
					Propofol group	1.5 mg/kg
Li et al.	2024	China	RCT	(1) (2) (3) (5) (6) (7) (8) (9) (10)	Ciprofol group	0.5 mg/kg
					Propofol group	2.0 mg/kg
Liao et al.	2023	China	RCT	(1) (2) (3) (9) (10)	Ciprofol group	0.4 mg/kg
					Propofol group	2.0 mg/kg
Liu et al.	2024	China	RCT	(2) (5) (6)	Ciprofol group	0.5 mg/kg
					Propofol group	2.0 mg/kg
Teng et al.	2021	China	RCT	(2) (6) (7) (8)	Ciprofol group	0.4 mg/kg
					Ciprofol group	0.5 mg/kg
					Propofol group	2.0 mg/kg
Zhang et al.	2024	China	RCT	(1) (2) (3) (4) (5) (6) (7) (8) (9)	Ciprofol group	0.3 mg/kg
					Propofol group	1.2 mg/kg
Zhou et al.	2024	China	RCT	(1) (8) (9)	Ciprofol group	0.3–0.5 mg/kg
					Propofol group	1.5–2.0 mg/kg

Abbreviations: (1) hypotension; (2) injection pain; (3) bradycardia; (4) respiratory depression; (5) induction time; (6) recovery time; (7) endoscopist satisfaction; (8) patient satisfaction; (9) hypoxemia; (10) nausea and vomiting; RCT, randomized controlled trial.

Initial doses are the doses reported by each trial. Supplemental bolus regimens were not consistently available and were not used in the dose-stratified analyses.

**TABLE 2:** Baseline patient characteristics in the included trials.

Author	Year	Comparison	BMI (kg/m^2^)	ASA (i/II/III)	Hypotension history	Diabetes history	Cardiovascular and cerebrovascular diseases	Mean age ±SD	Male/female	Number of patients	Endoscopy type (no. of patients)
Chen et al.	2023	Ciprofol group	23.41 ± 2.77	12/12/14	NA	NA	NA	52.03 ± 13.54	22/16	38	Gastroscopy (20), colonoscopy (18)
		Ciprofol group	22.65 ± 3.53	15/10/11	NA	NA	NA	45.58 ± 17.04	12/24	36	Gastroscopy (21), colonoscopy (15)
		Ciprofol group	22.99 ± 3.01	14/12/5	NA	NA	NA	47.45 ± 11.90	11/20	31	Gastroscopy (18), colonoscopy (13)
		Propofol group	22.16 ± 3.06	19/19/6	NA	NA	NA	43.55 ± 16.19	18/26	44	Gastroscopy (31) Colonoscopy (13)
Chen et al.	2022	Ciprofol group	25.22 ± 10.12	NA	NA	NA	NA	41.22 ± 11.63	22/25	47	Gastrointestinal endoscopy (47)
		Propofol group	23.46 ± 3.43	43/6/0	NA	NA	NA	43.20 ± 12.29	17/32	49	Gastrointestinal endoscopy (49)
Gao et al.	2024	Ciprofol group	23.4 ± 3.0	16/66/0	NA	NA	NA	52.50 ± 10.50	34/48	82	Colonoscopy (82)
		Propofol group	23.7 ± 3.0	20/62/0	NA	NA	NA	53.50 ± 9.50	32/50	82	Colonoscopy (82)
He et al.	2024	Ciprofol group	23.7 ± 2.9	29/81/0	17/93	5/105	1/109	48.00 ± 11.20	54/56	110	Colonoscopy (110)
		Propofol group	23.8 ± 2.7	36/76/0	16/96	5/107	2/110	49.00 ± 9.70	46/66	112	Colonoscopy (112)
Li et al.	2022	Ciprofol group	23.2 ± 2.5	115/29/0	NA	NA	NA	43.8 ± 11.80	55/89	144	Gastroscopy (15), colonoscopy (129)
		Propofol group	23.4 ± 2.6	118/27/0	NA	NA	NA	44.1 ± 11.30	63/82	145	Gastroscopy (15) Colonoscopy (130)
Li et al.	2024	Ciprofol group	23.19 ± 3.01	70/38/0	44/64	19/89	4/104	46.36 ± 11.20	48/60	108	Gastroscopy (108)
		Propofol group	23.42 ± 2.79	71/38/0	41/68	23/86	10/99	47.34 ± 12.33	49/60	109	Gastroscopy (109)
Liao et al.	2023	Ciprofol group	23.07 ± 2.28	79/106/0	NA	NA	NA	44.98 ± 11.74	87/98	185	Gastroscopy (99), gastrointestinal endoscopy (86)
		Propofol group	23.13 ± 2.23	62/121/0	NA	NA	NA	45.35 ± 11.12	77/106	183	Gastroscopy (94), gastrointestinal endoscopy (89)
Liu et al.	2024	Ciprofol group	23.00 ± 2.86	NA	NA	NA	NA	45.63 ± 10.07	13/17	30	Gastroscopy (30)
		Propofol group	23.69 ± 2.62	NA	NA	NA	NA	45.00 ± 12.96	18/12	30	Gastroscopy (30)
Teng et al.	2021	Ciprofol group	NA	25/6/0	NA	NA	NA	46.20 ± 13.00	12/19	31	Colonoscopy (31)
		Ciprofol group	NA	25/7/0	NA	NA	NA	47.50 ± 15.20	14/18	32	Colonoscopy (32)
		Propofol group	NA	25/6/0	NA	NA	NA	48.40 ± 13.70	16/15	31	Colonoscopy (31)
Zhang et al.	2024	Ciprofol group	23.40 ± 3.3	75/18/0	18/75	4/89	17/76	54.00 ± 11.10	52/41	93	Gastrointestinal endoscopy (93)
		Propofol group	23.30 ± 3.2	69/23/0	27/65	6/86	10/82	51.60 ± 11.10	52/40	92	Gastrointestinal endoscopy (92)
Zhou et al.	2024	Ciprofol group	NA	NA	NA	NA	NA	48.02 ± 12.05	52/68	120	Gastroscopy (38), colonoscopy (16), Gastrointestinal endoscopy (66)
		Propofol group	NA	NA	NA	NA	NA	48.72 ± 9.97	66/54	120	Gastroscopy (34), colonoscopy (18), Gastrointestinal endoscopy (68)

Abbreviations: NA, not available; BMI, body mass index; ASA, american society of anesthesiologists; SD, standard deviation.

Risk-of-bias judgments are presented in Figure 2. Two of the 11 trials were rated at high risk for selective reporting because important outcomes, including recovery or induction time, were not reported (Liao et al., 2023; Zhou et al., 2024). The remaining domains were predominantly judged low risk, although the overall certainty of the evidence should still be tempered by the limited number of trials and the fact that all studies were from China. Certainty of the evidence was assessed with the GRADE approach and is summarised in Supplementary Table 3. Certainty was moderate for hypotension, injection pain, bradycardia, respiratory depression, hypoxemia, and nausea and vomiting; low for recovery time, physician satisfaction, and patient satisfaction; and very low for induction time. Certainty was reduced mainly because of statistical heterogeneity, imprecision, and suspected publication bias for the two primary outcomes.

**FIGURE 2:**
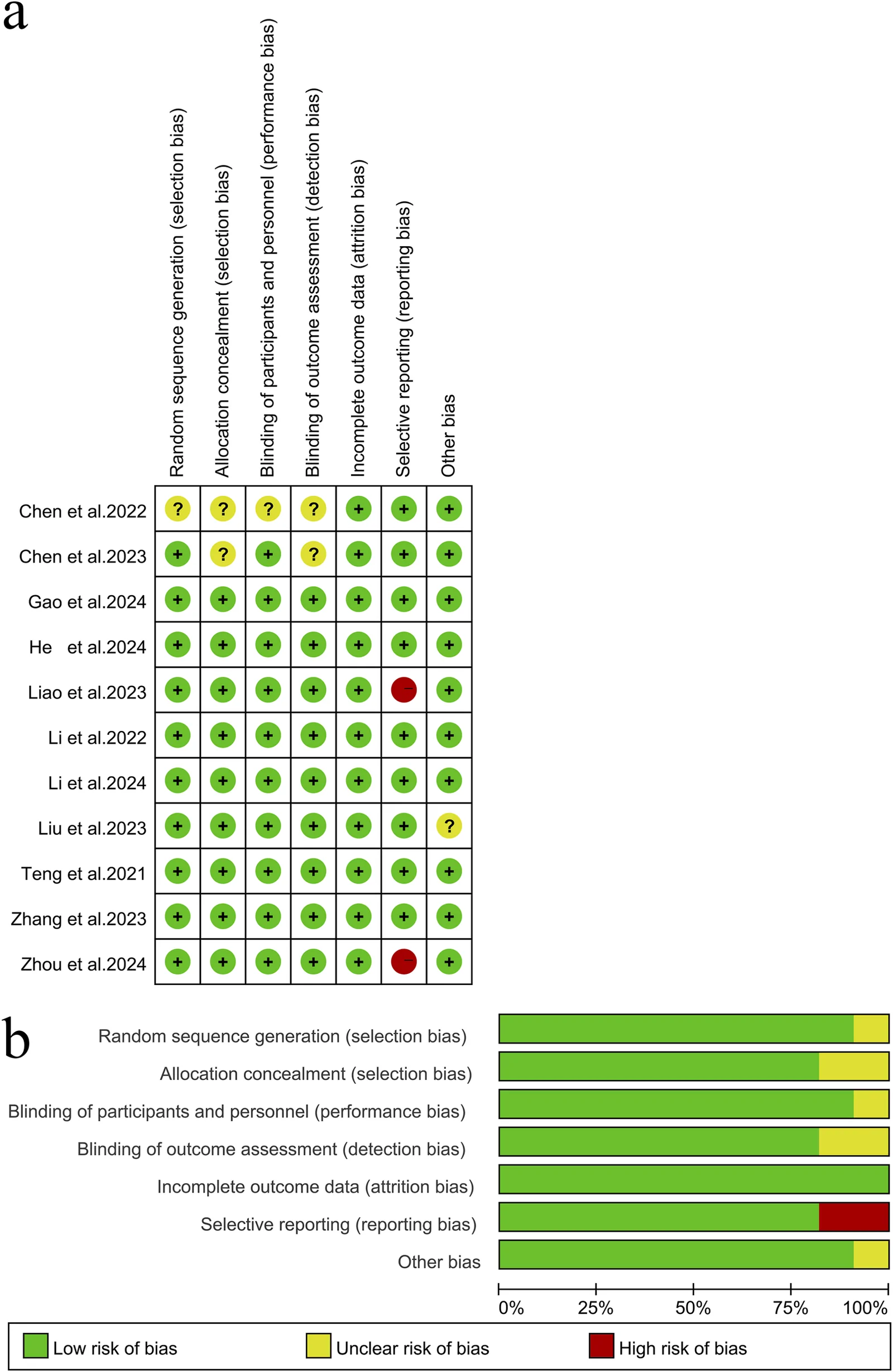
Risk-of-bias assessment for the included randomized trials. **(a)** Domain-level judgments for each trial. **(b)** The proportion of judgments classified as low, unclear, or high risk across domains.

### Recovery time

Recovery time was available from nine studies (Li et al., 2022; Teng et al., 2021; Li et al., 2024; Chen et al., 2023; Zhang et al., 2024; Chen et al., 2022; Gao et al., 2024; He et al., 2024; Liu Y. et al., 2024). Between-study inconsistency was high (I^2^ = 87.36%, P < 0.001). The pooled estimate favored propofol by less than 1 minute: recovery was 0.83 min longer with ciprofol (95% CI 0.16 to 1.49; P = 0.01; Figure 3a).

**FIGURE 3:**
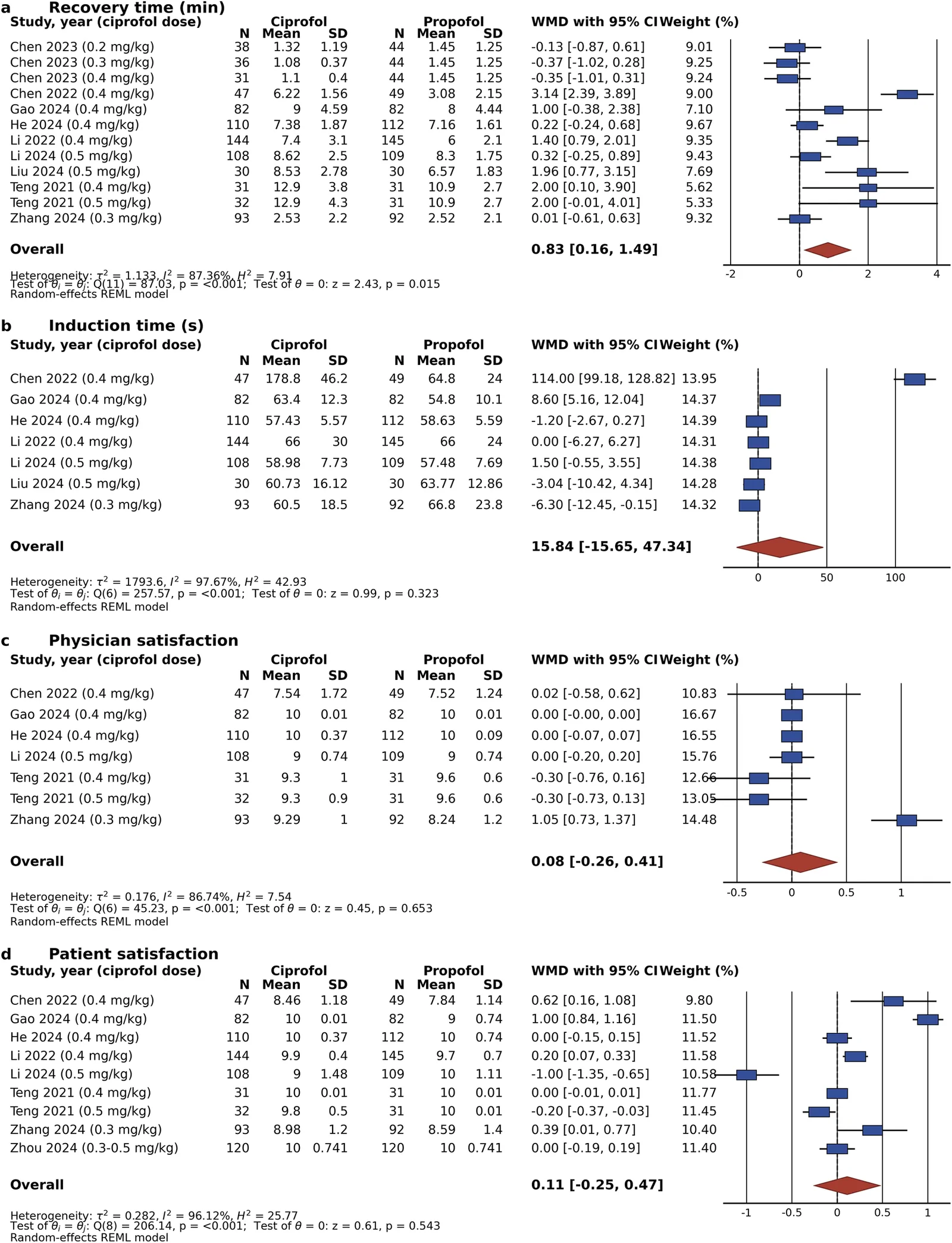
Forest plots comparing ciprofol with propofol for **(a)** recovery time, **(b)** induction time, **(c)** physician satisfaction, and **(d)** patient satisfaction during painless gastrointestinal endoscopy. In each forest plot, the square represents an individual study estimate and its size reflects study weight; the horizontal line is the 95% confidence interval, and the diamond summarizes the pooled weighted mean difference.

Removing one study at a time did not materially change the estimate, although this stability does not resolve the high heterogeneity. Egger’s test did not indicate clear funnel-plot asymmetry (P = 0.18; Supplementary Table 2). Trim-and-fill added no studies and returned the same estimate (WMD 0.83, 95% CI 0.16–1.49); because this procedure is model-dependent and only a small number of studies was available, it should not be interpreted as excluding missing-study bias.

Meta-regression did not establish a clear explanation for the heterogeneity. In dose-stratified analyses, recovery was longer with ciprofol at 0.4 mg/kg (*P* = 0.01) and 0.5 mg/kg (*P* = 0.04), whereas the estimates at 0.2 mg/kg (*P* = 0.63) and 0.3 mg/kg (*P* = 0.16) were not statistically different from propofol (Table 3). These subgroup results are exploratory because several dose categories contained few datasets.

**TABLE 3:** Subgroup and meta-regression analyses for the primary outcomes.

Subgroup	No. of datasets	Recovery time WMD (95% CI), *P* value	Meta-regression *P* value	No. of studies	Induction time WMD (95% CI), *P* value	Meta-regression *P* value
Ciprofol dose (mg/kg)	​	​	0.54	​	​	0.38
0.2 mg/kg	1	−0.13 (−0.66, 0.40), *P* = 0.63	​	0	NA	​
0.3 mg/kg	2	−0.25 (−0.60, 0.10), *P* = 0.16	​	1	−6.30 (−12.44, −0.16), *P* = 0.04	​
0.4 mg/kg	7	1.19 (0.25, 2.13), *P* = 0.01	​	5	26.49 (−16.52, 69.50), *P* = 0.23	​
0.5 mg/kg	3	1.26 (0.04, 2.48), *P* = 0.04	​	2	0.67 (−2.77, 4.11), *P* = 0.70	​
Endoscopy type	​	​	0.39	​	​	0.49
Gastroscopy	4	1.69 (0.39, 2.99), *P* = 0.01	​	3	1.15 (−1.19, 3.49), *P* = 0.34	​
Colonoscopy	5	1.07 (0.33, 1.81), P = 0.005	​	3	2.49 (−3.77, 8.74), *P* = 0.44	​

Abbreviations: WMD, weighted mean difference; CI, confidence interval; NA, not available.

Recovery was also longer with ciprofol in both gastroscopy and colonoscopy subgroups (*P* = 0.01 and *P* < 0.001, respectively; Table 3).

### Induction time

Seven studies contributed induction-time data (Li et al., 2022; Li et al., 2024; Zhang et al., 2024; Chen et al., 2022; Gao et al., 2024; He et al., 2024; Liu Y. et al., 2024). Heterogeneity was extreme (I^2^ = 97.67%, P < 0.001). The pooled comparison did not show a statistically significant difference between ciprofol and propofol (WMD 15.84, 95% CI −15.65 to 47.34; P = 0.32; Figure 3b), and the wide interval reduces confidence in a precise null interpretation.

Leave-one-out analysis did not materially change the pooled estimate. Egger’s test suggested possible publication bias (*P* = 0.001; Supplementary Table 4). Trim-and-fill produced WMD 24.10 (95% CI −2.25 to 50.45), which remained statistically compatible with no difference. This result indicates possible sensitivity to missing studies.

The dose meta-regression was not informative. At 0.4 and 0.5 mg/kg, induction was numerically longer with ciprofol but not statistically different from propofol (*P* = 0.23 and *P* = 0.70). At 0.3 mg/kg, induction was shorter with ciprofol (*P* = 0.04; Table 3).

In the endoscopy-type subgroups, induction was numerically longer with ciprofol in both gastroscopy and colonoscopy, but neither comparison was statistically significant (*P* = 0.34 and *P* = 0.44, respectively; Table 3).

### Satisfaction outcomes

Physician satisfaction was reported in six studies (Teng et al., 2021; Li et al., 2024; Zhang et al., 2024; Chen et al., 2022; Gao et al., 2024; He et al., 2024). Heterogeneity was very high (I^2^ = 86.74%, P < 0.001), and the pooled comparison did not differ (WMD 0.08, 95% CI −0.26 to 0.41; P = 0.67; Figure 3c). The estimate should therefore be viewed cautiously.

Eight studies reported patient satisfaction (Li et al., 2022; Teng et al., 2021; Li et al., 2024; Zhang et al., 2024; Zhou et al., 2024; Chen et al., 2022; Gao et al., 2024; He et al., 2024). Heterogeneity was similarly high (I^2^ = 96.12%, P < 0.001). No statistically significant difference was detected (WMD 0.11, 95% CI −0.25 to 0.47; P = 0.54; Figure 3d). The result is compatible with broadly similar satisfaction, but it does not establish equivalence.

## Adverse events

Across studies reporting hypotension, heterogeneity was low (I^2^ = 10.99%, P = 0.35). Hypotension was less frequent with ciprofol than with propofol (RR 0.81, 95% CI 0.72 to 0.90; P < 0.001; Supplementary Table 2).

The injection-pain analysis showed moderate heterogeneity (I^2^ = 58.76%, P = 0.004). Ciprofol was associated with less injection pain than propofol (RR 0.13, 95% CI 0.08 to 0.22; P < 0.001; Supplementary Table 2).

For respiratory depression, heterogeneity was minimal (I^2^ < 0.1%, P = 0.42), and the event was less frequent with ciprofol (RR 0.47, 95% CI 0.27 to 0.83; P = 0.009; Supplementary Table 2).

Hypoxemia also showed no statistically significant heterogeneity (I^2^ = 36.08%, P = 0.17). The pooled estimate favored ciprofol (RR 0.64, 95% CI 0.42 to 0.96; P = 0.03; Supplementary Table 2).

## Discussion

This review compared ciprofol and propofol for painless gastrointestinal endoscopy, with initial ciprofol dose examined as a potential modifier. Overall, induction time and satisfaction did not differ statistically, while recovery was 0.83 min longer with ciprofol. Ciprofol was associated with lower reported rates of hypotension, injection pain, respiratory depression, and hypoxemia. Longer recovery was observed at 0.4 and 0.5 mg/kg, but dose groups were small. All trials were conducted in China, and several outcomes were highly heterogeneous; the findings therefore require cautious, hypothesis-generating interpretation.

Induction time was not statistically different between the drugs, whereas ciprofol prolonged recovery by 0.83 min. Although the confidence interval excluded zero, a difference of about 50 s may have limited clinical relevance in many endoscopy units; its operational importance could nevertheless be greater where recovery capacity is constrained. The result is also influenced by heterogeneity and incomplete reporting of supplemental doses and recovery protocols. It is broadly consistent with the recent ciprofol-propofol review by Yu et al. (Yu et al., 2025). The agents share GABA-mediated sedative effects (Bian et al., 2021; Liu L. et al., 2024), but recovery may vary with central nervous system depression and drug disposition (Miller’s basics of anesthesia, 2024). The reported elimination half-life of ciprofol is somewhat longer than that of propofol (Li et al., 2021), while distribution, clearance, protein binding, supplemental dosing, and recovery definitions may also contribute (Liu Y. et al., 2024). The apparent pattern of shorter recovery at 0.2–0.3 mg/kg and longer recovery at 0.4–0.5 mg/kg is therefore best treated as exploratory rather than as a confirmed dose-response relationship (Bian et al., 2021; Guo et al., 2025).

Satisfaction depends on procedural conditions, cooperation, analgesia, depth of sedation, additional medication, and the timing of assessment (Zhang et al., 2024). We found no statistically significant difference in physician or patient satisfaction between groups (Yang et al., 2025; Wang et al., 2025; Liu J. et al., 2024). Similar sedation success and rapid onset may help explain the close estimates, but satisfaction is subjective and can also reflect expectations, cultural context, and when the questionnaire is administered. These results support comparable reported experiences, not interchangeability in every clinical setting.

Our pooled results suggested fewer hypotension and hypoxemia events with ciprofol than with propofol, but the result should be interpreted in light of how each trial defined and recorded adverse events. Differences in dose and pharmacology may contribute: ciprofol is commonly administered at a lower dose because of its greater potency (Zhou J. et al., 2025; Currò et al., 2024), and hemodynamic effects may also reflect vascular tone and myocardial contractility. Experimental and clinical evidence has described possible effects on endothelial nitric oxide synthase and L-type calcium channels (Chen et al., 2025), while the available clinical study reports the hemodynamic comparison in another procedural setting (Yu et al., 2025). Both agents act through GABA receptors, but differing receptor-subunit preferences have been proposed as one explanation for variation in respiratory effects (Zhou J. et al., 2025). The proposed receptor selectivity may affect respiratory-center suppression (Liao et al., 2022). The lower dose and the properties of the major metabolite may also influence respiratory safety (Zhou J. et al., 2025; Bian et al., 2021). These mechanisms remain plausible explanations rather than findings directly established by this meta-analysis.

Injection pain is a common limitation of propofol induction. In our analysis, pain was less frequent with ciprofol (RR 0.13, 95% CI 0.08 to 0.22; P < 0.001) (Ding et al., 2022). The concentration of unbound drug in the aqueous phase may affect local irritation, and ciprofol has been described as having a lower aqueous-phase concentration (Liao et al., 2023). Formulation, lipid solubility, and direct vascular stimulation may all influence the experience (Lu et al., 2023; Chen et al., 2023). These pharmacologic considerations are consistent with the observed association but do not eliminate the possibility that pain assessment and injection technique differed across trials.

Several recent meta-analyses have addressed ciprofol and propofol in endoscopic sedation (Liu J. et al., 2024), and another review reported that study eligibility and age may influence induction and recovery estimates (Cheng et al., 2025). Our analysis was narrower in one respect—it stratified initial ciprofol doses from 0.2 to 0.5 mg/kg and considered recovery and induction together—but the small subgroups, high heterogeneity, and inconsistent supplemental-dose reporting prevent identification of an optimal regimen.

Substantial heterogeneity affected several continuous outcomes, with I^2^ above 90% for recovery, induction, and satisfaction. Neither subgroup analysis nor meta-regression identified a definitive explanation. Dose and endoscopy type explained part of the recovery pattern, but age, ASA class, opioid co-administration, supplemental boluses, sedation depth, recovery criteria, and outcome definitions may also have contributed. Incomplete reporting prevented age- and ASA-based analyses. Although leave-one-out analyses did not suggest that one study drove the findings, they do not remove between-study differences. The pooled estimates should therefore be interpreted with reduced certainty and limited generalizability.

Propofol is established, widely available, and generally less expensive, whereas ciprofol is newer and may impose a larger cost burden (Wen et al., 2023). The present analysis did not include formal cost-effectiveness data. Ciprofol’s potentially gentler hemodynamic profile may be relevant to patients with reduced physiologic reserve, including older adults, but this interpretation requires population-specific evidence (Chen et al., 2024). Continuous-infusion data in older patients also suggest feasibility in selected settings (Guo et al., 2025). Some reviews have proposed additional safety or metabolic advantages, but these claims were not directly evaluated here (Lu et al., 2023). Obesity and other comorbidities can alter sedation risk, and the lower event rates observed in this analysis should not be generalized beyond the included trial populations (Xie et al., 2025). Thus, ciprofol may be a useful alternative for selected patients, but availability, cost, dose selection, and local monitoring resources remain important.

Several limitations should be considered. First, the search covered only PubMed and Embase, English-language reports, and reference-list screening; grey literature was not systematically searched, so unpublished or non-indexed evidence may have been missed. Second, all included trials were conducted in China, so the results apply primarily to Chinese populations and healthcare settings and require confirmation in other countries. Third, heterogeneity was substantial for several outcomes, and age, ASA class, co-administered opioids, supplemental dosing, recovery protocols, and procedure characteristics could not be examined adequately. Fourth, definitions of hypotension, respiratory depression, and hypoxemia differed among trials, so pooled safety estimates may partly reflect measurement differences. Fifth, the dose subgroups were small, and reported initial doses should not be interpreted as pharmacologically equivalent doses of the two drugs. Sixth, uncommon but important outcomes such as aspiration, airway intervention, unplanned admission, and recall were not assessed. Finally, certainty of the evidence was assessed with GRADE and was moderate at best (Supplementary Table 3), so the findings remain hypothesis-generating. Larger multicenter and international RCTs with standardized outcome definitions and complete dosing information are needed.

## Conclusion

Among adults undergoing painless gastrointestinal endoscopy, ciprofol produced broadly similar induction and satisfaction outcomes to propofol and was associated with lower reported rates of several adverse events and injection pain. Recovery was statistically longer by 0.83 min, but the clinical relevance of this small difference is uncertain. The evidence is restricted to Chinese RCTs, includes substantial heterogeneity and possible publication bias, uses non-uniform adverse-event definitions, and does not address several uncommon but important outcomes. The results should therefore be viewed as hypothesis-generating. Larger international, multicenter trials with standardized safety definitions, complete dosing information, and patient-centered recovery outcomes are needed.

## Data Availability

The original contributions presented in the study are included in the article/Supplementary Material, further inquiries can be directed to the corresponding author.
