# Supplementary material for: Ciprofol versus propofol for painless gastrointestinal endoscopy: a dose-stratified systematic review and meta-analysis
Source: Front Pharmacol. 2026 Sep 25;17:1977897. doi: 10.3389/fphar.2026.1977897 (PMC13649421; doi:10.3389/fphar.2026.1977897)
Supplement: Supplementary file 2 [file DataSheet2.docx]

SUPPLEMENTARY MATERIALS

**Ciprofol versus propofol for painless gastrointestinal endoscopy: a dose-stratified systematic review and meta-analysis**

SYSTEMATIC REVIEW AND META-ANALYSIS

This supplementary material comprises detailed search strategies for PubMed and Embase, comprehensive summaries of adverse-event meta-analyses, leave-one-out sensitivity analyses for recovery and induction times, funnel plots for publication-bias assessment, and a GRADE summary of findings.

11 randomized controlled trials • 2,084 adults • Dose-stratified analyses

3 Supplementary Tables · 4 Supplementary Figures · 10 pages

**Contents**

**Supplementary tables and figures**

**SUPPLEMENTARY TABLES**

| Supplementary Table 1. Search strategies for PubMed and Embase. | **3** |
| --- | --- |
| Supplementary Table 2. Summary of adverse events. | **4** |
| Supplementary Table 3. Summary of findings of certainty of the evidence. | **5** |

**SUPPLEMENTARY FIGURES**

| Supplementary Figure 1. Leave-one-out sensitivity analysis for recovery time. | 7 |
| --- | --- |
| Supplementary Figure 2. Funnel plot for recovery time. | **8** |
| Supplementary Figure 3. Leave-one-out sensitivity analysis for induction time. | **9** |
| Supplementary Figure 4. Funnel plot for induction time. | **10** |

Supplementary Table 1. Search strategies for PubMed and Embase.

| **Database** | **Search strategy** |
| --- | --- |
| **PubMed (101)** | ("Ciprofol" OR "HSK3486" OR “Cipepofol”) AND ("Propofol"[Mesh] OR "Propofol" OR "Diprivan" OR “Disoprofol” OR “Disoprivan” OR "Aquafol" OR "Fresofol" OR "Ivofol" OR "Recofol") |
| **Embase (109)** | ('cipepofol'/exp OR ‘ciprofol’ OR ‘cipepofol’ OR ‘HSK3486’) AND ('propofol'/exp OR ‘Propofol’ OR ‘Diprivan’ OR ‘Disoprofol’ OR ‘Disoprivan’ OR ‘Aquafol’ OR ‘Fresofol’ OR ‘Ivofol’ OR ‘Recofol’) |

***Note. Database-specific syntax and controlled vocabulary are reproduced as used in the search strategy.***

Supplementary Table 2. Summary of adverse events.

| **Adverse Event** | **Number of Datasets** | **I² (%) and Cochran Q *P* value** | **Ciprofol vs. Propofol RR (95% CI), *P* value** |
| --- | --- | --- | --- |
| Incidence of hypotension | 7 | 10.99%, P = 0.35 | 0.81 (0.72, 0.90), P < 0.001 |
| Injection pain | 13 | 58.76%, P = 0.004 | 0.13 (0.08, 0.22), P < 0.001 |
| Bradycardia | 6 | 0.00%, P = 0.68 | 0.86 (0.58, 1.27), P = 0.44 |
| Respiratory depression | 6 | < 0.1%, P = 0.42 | 0.47 (0.27, 0.83), P = 0.009 |
| Hypoxemia | 6 | 36.08%, P = 0.17 | 0.64 (0.42, 0.96), P = 0.03 |
| Nausea and vomiting | 7 | 0.00%, P = 0.76 | 0.82 (0.57, 1.19), P = 0.31 |

*RR, risk ratio; CI, confidence interval; NA, not available.*

Supplementary Table 3. Summary of findings of certainty of the evidence (GRADE).

| **Outcome** | **Risk of biasᵃ** | **Inconsistencyᵇ** | **Indirectnessᶜ** | **Imprecisionᵈ** | **Publication biasᵉ** | **Total Downgrade** | **Certainty** |
| --- | --- | --- | --- | --- | --- | --- | --- |
| Recovery time | 0 | -1 | 0 | 0 | -1 | 2 | Low |
| Induction time | 0 | -1 | 0 | -1 | -1 | 3 | Very low |
| Physician satisfaction | 0 | -1 | 0 | -1 | 0 | 2 | Low |
| Patient satisfaction | 0 | -1 | 0 | -1 | 0 | 2 | Low |
| Hypotension | 0 | 0 | 0 | -1 | 0 | 1 | Moderate |
| Injection pain | 0 | -1 | 0 | 0 | 0 | 1 | Moderate |
| Bradycardia | 0 | 0 | 0 | -1 | 0 | 1 | Moderate |
| Respiratory depression | 0 | 0 | 0 | -1 | 0 | 1 | Moderate |
| Hypoxemia | 0 | 0 | 0 | -1 | 0 | 1 | Moderate |
| Nausea and vomiting | 0 | 0 | 0 | -1 | 0 | 1 | Moderate |

a. **Risk of Bias**
Assessed using the ROB tool to evaluate methodological quality of RCTs.
Rated down one level for **serious risk of bias**. The pooled effect estimate is dominated by studies (>55% weight) assessed as high risk of bias using ROB.
Rated down two levels for **very serious risk of bias**. The majority of evidence comes from studies with extremely serious limitations in multiple domains of the **ROB** tool (e.g., lack of blinding, failure of allocation concealment, and critical missing outcome data), which strictly undermines confidence in the result.

b. **Inconsistency**
Measured using I² statistics and overlap of confidence intervals across studies.
Rated down one level if I² > 50% with *P* < 0.1 and point estimates show conflicting directions.

Rated down two levels for **very serious inconsistency**. The heterogeneity is extremely high (I² > 90%), and the results across studies are diametrically opposed (substantial benefit vs. substantial harm) with non-overlapping confidence intervals, which could not be explained by a priori subgroup hypotheses.

c. **Indirectness**
Evaluates whether PICO elements match the target clinical question.
Rated down one level for population, intervention, or outcome mismatches.
Rated down two levels for **very serious indirectness**. The available evidence assesses a surrogate outcome rather than the patient-important outcome of interest, and the correlation between the surrogate and the clinical outcome is uncertain. In this review all included trials were conducted in China. Because the target population was adults undergoing painless gastrointestinal endoscopy and no evidence indicated that the sedation effects of the two drugs differ by country or ethnicity, this geographical restriction was judged to affect applicability (and is addressed in the Limitations) rather than to constitute indirectness; no level was therefore deducted for indirectness.

d. **Imprecision**
Assessed based on confidence intervals and sample size relative to optimal information size.
Imprecision was assessed based on the relationship between 95% confidence intervals (CIs), clinical decision thresholds, and the optimal information size (OIS). We evaluated whether the 95% CIs crossed the null effect or the minimal important difference (MID). Even if thresholds were not crossed, imprecision was further scrutinized using OIS calculation. For binary outcomes, OIS was determined assuming a type I error of 0.05, 80% power, a control group event rate derived from the pooled studies, and a conservative relative risk reduction (RRR) of 25%. For continuous outcomes, a total sample size of 800 participants was adopted as the threshold for adequacy.

Rated down two levels for **very serious imprecision**. The 95% CI is very wide, including both substantial benefit and substantial harm.

e. **Publication Bias**
Evaluated using funnel plot symmetry and Egger’s test.

Rated down one level for strongly suspected publication bias. The funnel plot shows evident asymmetry, suggesting that small studies with negative results are missing. In this review, funnel plots and Egger’s test were performed only for recovery time and induction time (the two outcomes with the largest number of datasets); for outcomes with few datasets, publication bias could not be assessed reliably and no level was deducted. All outcomes started at high certainty because every included study was a randomised trial. The total downgrade is the sum of the levels deducted: high (no downgrade), moderate (one), low (two), or very low (three or more).

Supplementary Figure 1. Leave-one-out sensitivity analysis for recovery time.

Supplementary Figure 2. Funnel plot for recovery time.

Supplementary Figure 3. Leave-one-out sensitivity analysis for induction time.

Supplementary Figure 4. Funnel plot for induction time.
